# Supplementary material for: Altered orbitofrontal sulcogyral patterns in gambling disorder: a multicenter study
Source: Transl Psychiatry. 2019 Aug 5;9:186. doi: 10.1038/s41398-019-0520-8 (PMC6683128; doi:10.1038/s41398-019-0520-8)
Supplement: Supplementary file 1 — supplemental material [file 41398_2019_520_MOESM1_ESM.docx]

**Supplementary Materials**

**Altered orbitofrontal sulcogyral patterns in gambling disorder: a multicenter study**

Yansong Li^1,2*^, Zixiang Wang^1,2*^, Isabelle Boileau^3^, Jean-Claude Dreher^4^, Sofie Gelskov^5^, Alexander Genauck^6^, Juho Joutsa^7^, Valtteri Kaasinen^7^, José C Perales^8^, Nina Romanczuk-Seiferth^6^, Cristian M Ruiz de Lara^8^, Hartwig R Siebner^5^, Ruth J van Holst^9^, Tim van Timmeren^9^, Guillaume Sescousse^10^

^1^ Competition, Status and Social Neuroscience Lab, Department of Psychology, School of Social and Behavioral Sciences, Nanjing University, Nanjing, China

^2^ Institute for Brain Sciences, Nanjing University, Nanjing, China

^3^ Campbell Family Mental Health Research Institute and Research Imaging Centre, Centre for Addiction and Mental Health, Toronto, Canada

^4^ ‘Neuroeconomics Laboratory, Institut des Sciences Cognitives Marc Jeannerod, CNRS UMR 5229, Bron, France

^5^ Danish Research Centre for Magnetic Resonance, Centre for Functional and Diagnostic Imaging and Research, Copenhagen University Hospital Hvidovre, Hvidovre, Denmark

^6^ Department of Psychiatry and Psychotherapy, Charité – Universitätsmedizin Berlin, Corporate member of Freie Universität Berlin, Humboldt-Universität zu Berlin, and Berlin Institute of Health, Berlin, Germany

^7^ Division of Clinical Neurosciences, University of Turku and Turku University Hospital, Turku, Finland

^8^ Department of Experimental Psychology, Mind, Brain and Behavior Research Center (CIMCYC), University of Granada, Granada, Spain

^9^ Amsterdam UMC, Department of Psychiatry, Amsterdam Institute for Addiction Research, University of Amsterdam, Amsterdam, The Netherlands

^10^ Lyon Neuroscience Research Center - INSERM U1028 - CNRS UMR5292, PSYR2 Team, University of Lyon, Lyon, France

**Supplementary Table 1.** Information on individual studies.

|  | *Boileau et al., 2014* | *Gelskov et al., 2016* | *Genauck et al., 2018* | *Joutsa et al., 2011* | *Ruiz de Lara et al., 2018* | *Romanczuk-Seiferth et al., 2015* | *Sescousse et al., 2013* | *Sescousse et al., 2016* | *van Timmeren et al., 2017* |
| --- | --- | --- | --- | --- | --- | --- | --- | --- | --- |
| Number of pathological gamblers (Number excluded) | 13 (1) | 15 (0) | 22 (4) | 12 (0) | 26 (5) | 19 (2) | 20 (0) | 22 (0) | 28 (0) |
| Number of healthy controls (Number excluded) | 11 (1) | 15 (0) | 23 (1) | 12 (2) | 27 (2) | 17 (2) | 19 (1) | 22 (0) | 23 (1) |
| Manufacturer and Model of scanner | GE Signa | Siemens  Magnetom Tim Trio | Siemens  Magnetom Tim Trio | Philips Gyro-  scan Intera | Siemens Magnetom Tim Trio Syngo MR B17 | Siemens Magnetom Tim Trio | Siemens Sonata | Siemens Magnetom Tim Trio | Philips Intera |
| Field strength (Tesla) | 1.5 | 3 | 3 | 1.5 | 3 | 3 | 1.5 | 3 | 3 |
| Number of channels of head coil | 8 | 8 | 12 | 8 | 32 | 12 | 8 | 32 | 8 |
| Repetition Time (ms) | 8.9–12 | 1540 | 1900 | 25 | 2300 | 1570 | 1970 | 2300 | 1530 |
| Echo Time (ms) | 5.3–15 | 3.9 | 2.52 | 4.6 | 3.1 | 2.7 | 3.93 | 3.03 | 4.2 |
|  |  |  |  |  |  |  |  |  |  |
| Sequence | T1-weighted spoiled gradient recalled acquisition | T1-weighted  MP-RAGE | T1-weighted  MP-RAGE | T1-weighted fast field echo | T1-weighted MP-RAGE | T1-weighted MP-RAGE | T1-weighted MP-RAGE | T1-weighted MP-RAGE | T1-weighted  MP-RAGE |

**Supplementary Table 2.** Orbitofrontal Cortex (OFC) sulcogyral pattern distribution in pathological gamblers by studies.

|  | *Boileau et al., 2014* | *Gelskov et al., 2016* | *Genauck et al., 2018* | *Joutsa et al., 2011* | *Ruiz de Lara et al., 2018* | *Romanczuk-Seiferth et al., 2015* | *Sescousse et al., 2013* | *Sescousse et al., 2016* | *van Timmeren et al., 2017* |
| --- | --- | --- | --- | --- | --- | --- | --- | --- | --- |
| Left OFC sulcogyral pattern, n (%) | | | | | | | | | |
| Type I | 5 (42) | 8 (53) | 8 (44) | 4 (33) | 8 (38) | 8 (47) | 5 (25) | 8 (36) | 11 (39) |
| Type II | 5 (42) | 6 (40) | 9 (50) | 6 (50) | 9 (43) | 5 (29) | 11 (55) | 12 (55) | 13 (46) |
| Type III | 1 (8) | 1 (7) | 1 (6) | 2 (17) | 3 (14) | 4 (24) | 4 (20) | 2 (9) | 1 (4) |
| Type IV | 1 (8) | 0 (0) | 0 (0) | 0 (0) | 1 (5) | 0 (0) | 0 (0) | 0 (0) | 3 (11) |
| Right OFC sulcogyral pattern, n (%) | | | | | | | | | |
| Type I | 5 (42) | 7 (47) | 7 (38) | 3 (25) | 11 (53) | 10 (59) | 10 (50) | 8 (36) | 14 (50) |
| Type II | 4 (33) | 7 (47) | 10 (56) | 7 (59) | 7 (33) | 5 (29) | 10 (50) | 9 (41) | 13 (46) |
| Type III | 2 (17) | 0 (0) | 0 (0) | 1 (8) | 3 (14) | 2 (12) | 0 (0) | 4 (19) | 1 (4) |
| Type IV | 1 (8) | 1 (6) | 1 (6) | 1 (8) | 0 (0) | 0 (0) | 0 (0) | 1 (4) | 0 (0) |

**Supplementary Table 3.** Orbitofrontal Cortex (OFC) sulcogyral pattern distribution in healthy controls by studies.

|  | *Boileau et al., 2014* | *Gelskov et al., 2016* | *Genauck et al., 2018* | *Joutsa et al., 2011* | *Ruiz de Lara et al., 2018* | *Romanczuk-Seiferth et al., 2015* | *Sescousse et al., 2013* | *Sescousse et al., 2016* | *van Timmeren et al., 2017* |
| --- | --- | --- | --- | --- | --- | --- | --- | --- | --- |
| Left OFC sulcogyral pattern, n (%) | | | | | | | | | |
| Type I | 4 (40) | 7 (47) | 11 (50) | 5 (50) | 14 (56) | 7 (47) | 7 (39) | 12 (55) | 7 (32) |
| Type II | 4 (40) | 7 (47) | 7 (32) | 3 (30) | 8 (32) | 5 (33) | 7 (39) | 6 (27) | 8 (36) |
| Type III | 2 (20) | 1 (6) | 4 (18) | 2 (20) | 2 (8) | 3 (20) | 4 (22) | 4 (18) | 5 (23) |
| Type IV | 0 (0) | 0 (0) | 0 (0) | 0 (0) | 1 (4) | 0 (0) | 0 (0) | 0 (0) | 2 (9) |
| Right OFC sulcogyral pattern, n (%) | | | | | | | | | |
| Type I | 5 (50) | 7 (47) | 13 (59) | 5 (50) | 11 (44) | 8 (53) | 11 (61) | 11 (50) | 13 (59) |
| Type II | 3 (30) | 8 (53) | 5 (23) | 4 (40) | 11 (44) | 6 (40) | 5 (27) | 7 (32) | 4 (18) |
| Type III | 2 (20) | 0 (0) | 4 (18) | 1 (10) | 3 (12) | 1 (7) | 1 (6) | 4 (18) | 4 (18) |
| Type IV | 0 (0) | 0 (0) | 0 (0) | 0 (0) | 0 (0) | 0 (0) | 1 (6) | 0 (0) | 1 (5) |

**Supplementary Table 4**. Distribution of OFC sulcogyral patterns and associated SOGS scores as a function of left/right hemisphere combination.

|  | Sulcogyral patterns | | Left | | | |
| --- | --- | --- | --- | --- | --- | --- |
|  |  |  | I | II | III | IV |
| Pathological gamblers | Right | I | N=33  (10.00 ± 4.51) | N=32  (10.13 ± 4.01) | N=8  (9.80 ± 4.21) | N=2  (9.50 ± 2.12) |
|  |  | II | N=28  (10.58 ± 3.65) | N=37  (10.15 ± 4.71) | N=5  (9.00 ± 4.85) | N=2  (11.50 ± 4.95) |
|  |  | III | N=3  (12.50 ± 2.12) | N=5  (9.60 ± 2.70) | N=5  (13.75 ± 3.30) | N=0 |
|  |  | IV | N=1  (14.00) | N=2  (10.50 ± 0.71) | N=1  (4.00) | N=1  (17.00) |
|  |  |  |  |  |  |  |
|  | Sulcogyral patterns | | Left | | | |
|  |  |  | I | II | III | IV |
| Healthy controls | Right | I | N=38  (0.41 ± 0.82) | N=29  (0.62 ± 1.13) | N=16  (0.27 ± 1.03) | N=1  (0.00) |
|  |  | II | N=26  (0.48 ± 0.95) | N=20  (0.33 ± 0.49) | N=5  (0.50 ± 1.00) | N=2  (0.00 ± 0.00) |
|  |  | III | N=9  (0.44 ± 1.01) | N=6  (0.33 ± 0.82) | N=5  (0.00 ± 0.00) | N=0 |
|  |  | IV | N=1  (0.00) | N=0 | N=1  (0.00) | N=0 |

*The numbers outside the brackets represent the numbers of participants for each left-right combination of the four sulcogyral patterns, while the numbers in brackets represent the mean ± standard deviation of SOGS scores.*

**References**

Boileau I, Payer D, Chugani B, Lobo D, Houle S, Wilson A, Warsh J, Kish S, Zack M (2014) In vivo evidence for greater amphetamine-induced dopamine release in pathological gambling: A positron emission tomography study with [11 C]-(+)-PHNO. Molecular Psychiatry 19:1305-1313.

Gelskov SV, Madsen KH, Ramsøy TZ, Siebner HR (2016) Aberrant neural signatures of decision-making: Pathological gamblers display cortico-striatal hypersensitivity to extreme gambles. NeuroImage 128:342-352.

Genauck A, Matthis C, Andrejevic M, Ballon L, Chiarello F, Duecker K, Heinz A, Kathmann N, Romanczuk-Seiferth N (2018) Neural correlates of cue-induced changes in decision-making distinguish subjects with gambling disorder from healthy controls. BioRxiv: <https://doi.org/10.1101/498725>.

Joutsa J, Saunavaara J, Parkkola R, Niemelä S, Kaasinen V. Extensive abnormality of brain white matter integrity in pathological gambling. Psychiatry Research: Neuroimaging 2011; 194(3): 340-346.

Romanczuk‐Seiferth N, Koehler S, Dreesen C, Wüstenberg T, Heinz A (2015) Pathological gambling and alcohol dependence: Neural disturbances in reward and loss avoidance processing. Addiction Biology 20:557-569.

Ruiz de Lara CM, Navas JF, Soriano-Mas C, Sescousse G, Perales JC (2018) Regional grey matter volume correlates of gambling disorder, gambling-related cognitive distortions, and emotion-driven impulsivity. International Gambling Studies 18:195-216.

Sescousse G, Barbalat G, Domenech P, Dreher J-C (2013) Imbalance in the sensitivity to different types of rewards in pathological gambling. Brain 136:2527-2538.

Sescousse G, Janssen LK, Hashemi MM, Timmer MHM, Geurts DEM, ter Huurne NP, Clark L, Cools R (2016) Amplified striatal responses to near-miss outcomes in pathological gamblers. Neuropsychopharmacology 41:2614-2623.

van Timmeren T, Jansen JM, Caan MWA, Goudriaan AE, Holst RJ (2017) White matter integrity between left basal ganglia and left prefrontal cortex is compromised in gambling disorder. Addiction Biology 22:1590-1600.
